# A Hand-book of Pathological Anatomy and Histology

**Published:** 1890-03

**Authors:** 


**A Hand-book of Pathological Anatomy and Histology.** By FRANCIS DELAFIELD, M. D., Professor of Pathology and Practical Medicine, College of Physicians and Surgeons, New York, and T. Mitchell Prudden, M. D., Director of the Laboratory of the Alumni Association of the College of Physicians and Surgeons, New York. Third edition, illustrated by 224 wood engravings, printed in black and colors. New York: William Wood & Co. 1889.

The objects which the authors have kept in view in preparing this third edition, are similar to those preceding. No better criterion is necessary to attest the favor which this work on pathology has received, than the fact that in a short space of time two large editions have been exhausted.

It is a work intended for the student and practitioner, being comprehensive for the former, and not too elementary for the latter.

It takes up the various procedures in pathology, as no other text-book does—fulfilling, therefore, a need heretofore left wanting.

Part I. is devoted to the method of making post-mortem examinations, giving in brief the points to be noted in external inspection, and the manner in opening and removing the internal organs. These authors follow the same rules laid down by Virchow in his manual on post-mortem examinations, but supplement them somewhat by the methods in vogue, to harden the different organs and to prepare them for microscopical examination.

The chapter on the examination of the bodies of new-born children is one that is worthy of consideration, especially from a medico-legal standpoint. Taylor's conclusions regarding the hydrostatic test of the lungs are appended, and show that too much care cannot be exercised by the physician confronted by such a case.

The chapter on general methods of presenting pathological specimens, and preparing them for study, will be of great service to laboratory students. The number of staining methods are, however, too limited, the carmine stains being entirely lost sight of.

Part II. takes up the changes in the circulation and composition of the blood, the degenerations, animal parasites and bacteria, inflammation and tumors. These subjects are all ably discussed though, perhaps, some of them are considered somewhat briefly. The classification of the infiltrations and degenerations differ slightly from other authors, a field in which differences of opinion still exists regarding these two allied processes.

The chapter on tumors is carefully and ably presented, the numerous excellent engravings helping to render this chapter, though irksome, interesting.

Part III. is devoted to the study of the morbid anatomy of the organs. In order are taken up: Diseases of the nervous system, respiratory system, vascular system, diseases of the abdominal organs, bones, joints, and muscles.

Part IV. treats of the lesions found in the general diseases, in poisoning, and in violent deaths. This, the concluding chapter, is of especial importance in its relation to legal questions. Too little stress is laid upon the conditions found in sudden deaths, as taught by many of our schools, and yet, in many cases, the physician has no more important decision to render than that of accidental or violent death. The introduction of this chapter is certainly a step towards educating medical men in one of the most important branches of medicine.

The work, on the whole, is to be highly commended. The high standing that the authors have taken in pathology in this country, insure for their work a confidence which few enjoy. The illustrations are all original, drawn by the authors themselves, and in accuracy and excellence can scarcely be surpassed.

The typography is all that is to be desired; paper, print, and binding are of the best. The publishers do not believe in the pernicious habit of having several antiquated catalogues bound in the body of the book. Those who have frequent recourse to the indexes will appreciate this happy omission. W. C. K.

